# Validation of advanced hybrid SPECT/CT system using dynamic anthropomorphic cardiac phantom

**DOI:** 10.1007/s12149-024-01966-w

**Published:** 2024-08-30

**Authors:** Elad Gelbart, Alexander Krakovich, Yigal Sherm, Gilad Rabin, Hagit Ratner, Mickey Scheinowitz, Ronen Goldkorn

**Affiliations:** 1 Faculty of Medical and Health Sciences, Tel-Aviv University, Tel-Aviv, Israel; 2 Department of Biomedical Engineering, Faculty of Engineering, Tel-Aviv University, Tel-Aviv, Israel; 3 GE HealthCare Molecular Imaging, Haifa, Israel; 4 Nuclear Cardiology Center, Lev Leviev Heart Institute, Sheba Medical Center, 52621 Ramat-Gan, Tel Hashomer, Israel

**Keywords:** Nuclear cardiology, SPECT, MBF, StarGuide, Anthropomorphic phantom

## Abstract

**Objective:**

Myocardial blood flow (MBF) assessment can provide incremental diagnostic and prognostic information and thus the validation of dynamic SPECT is of high importance. We recently developed a novel cardiac phantom for dynamic SPECT validation and compared its performance against the GE Discovery NM 530c. We now report its use for validation of a new hybrid SPECT/CT System featuring advanced cadmium zinc telluride (CZT) technology in a ring array detector design (StarGuide™, GE HealthCare).

**Methods:**

Our recently developed cardiac phantom with injected technetium-99m radiotracer was used to create physiological time activity curves (TACs) for the left ventricular (LV) cavity and the myocardium. The TACs allow the calculation of uptake rate (K1) and MBF. The StarGuide system was used to acquire and process the TACs, and these were compared to the TACs produced by the phantom and its mathematical model. Fifteen (15) experiments with different doses representing various MBF values were conducted, and a standard statistic tool was applied for significance.

**Results:**

The TACs produced by the StarGuide system had a significant correlation (*p* < 0.001) with the reference TACs generated by the phantom both for the LV (r = 0.94) and for the myocardium (r = 0.89). The calculated MBF difference between the system and the phantom was 0.14 ± 0.16 ml/min/g and the average relative absolute difference was 13.2 ± 8.1%. A coefficient of variance of ≤ 11% was observed for all MBF subranges. The regional uptake rate values were similar to the global one with a maximum difference of 5%.

**Conclusions:**

Our newly developed dynamic cardiac phantom was used for validation of the dynamic hybrid SPECT/CT CZT-based system (StarGuide™, GE). The accuracy and precision of the system for assessing MBF values were high. The new StarGuide system can reliably perform dynamic SPECT acquisitions over a wide range of myocardial perfusion flow rates.

## Introduction

Absolute myocardial blood flow (MBF), coronary flow reserve (CFR) and coronary flow capacity (CFC) provide incremental diagnostic and prognostic value over static myocardial perfusion imaging (MPI) [1–6]. Recently, dynamic single-photon emission computed tomography (SPECT) systems have reached the high accuracy level of positron emission tomography (PET) in assessing these important parameters [7–9]. This is attributed mainly to the development of the high sensitivity cadmium zinc telluride (CZT) cameras [10, 11] and is highly important given that SPECT is much more affordable and widely available.

As SPECT systems continue to advance, new tools are needed for the development and the validation of advanced systems and features. Various phantom models have been developed over the years for these purposes [12–18] but a phantom model which can simulate physiological and clinical behavior and allow fast and simple validation of SPECT systems is still in need. We previously developed an anthropomorphic beating phantom which is comprised of a unique cardiac insert placed in a commercial phantom torso and a set of pumps which allow injection and washout of a radiotracer [19, 20]. The phantom can mimic physiological behavior and produce time activity curves (TACs) for the left ventricular cavity and the myocardium using a mathematical model [19]. The produced TACs allow the validation of the dynamic capabilities of the nuclear camera. The uptake rate (K1) and the MBF can be calculated from the TACs using a net retention model [21, 22] and compared to K1 and MBF calculated by the SPECT system.

StarGuide is a new and advanced SPECT/CT 3D-ring CZT-based system developed by GE HealthCare, Haifa, Israel. It utilizes 12 CZT detectors, 7.25 mm-thick each. The detectors convert gamma ray photons into digital signals for accurate identification of the event location and energies emitted. The system design enables closer to the patient acquisition and focused scanning modes. The detectors can sweep to focus on a prespecified volume for better efficiency. The system can also detect a wide energy range, from 40 to 500 keV, and multiple isotopes. Several studies have already evaluated its capabilities [23, 24] but its dynamic cardiac validation is still at an early stage [25]. Our team previously validated the MBF measurement accuracy of the nuclear camera Discovery NM 530c (GE HealthCare, Haifa, Israel) using the phantom [19, 20]. We hypothesized that our anthropomorphic phantom could also be used to validate the dynamic measurement of the advanced SPECT/CT 3D-ring CZT-based system.

### Methods

Fifteen (n = 15) experiments at various simulated MBF values (range 0.5–2 ml/min/g, up to 3 repetitions) were conducted to compare the StarGuide dynamic parameters to those of the phantom. For each experiment, two syringes containing saline with Tc-99m were prepared: one syringe for the LV compartment and one for the myocardium. The activities were measured in a dose calibrator (CRC-55t, Capintec, NJ, USA). The dose calibrator was also used to measure the remaining activity in the empty syringes and the activity at each compartment at the end of the scan by taking 10 mL samples from the LV and the myocardium using two additional syringes.

### Phantom

**Fig. 1:**
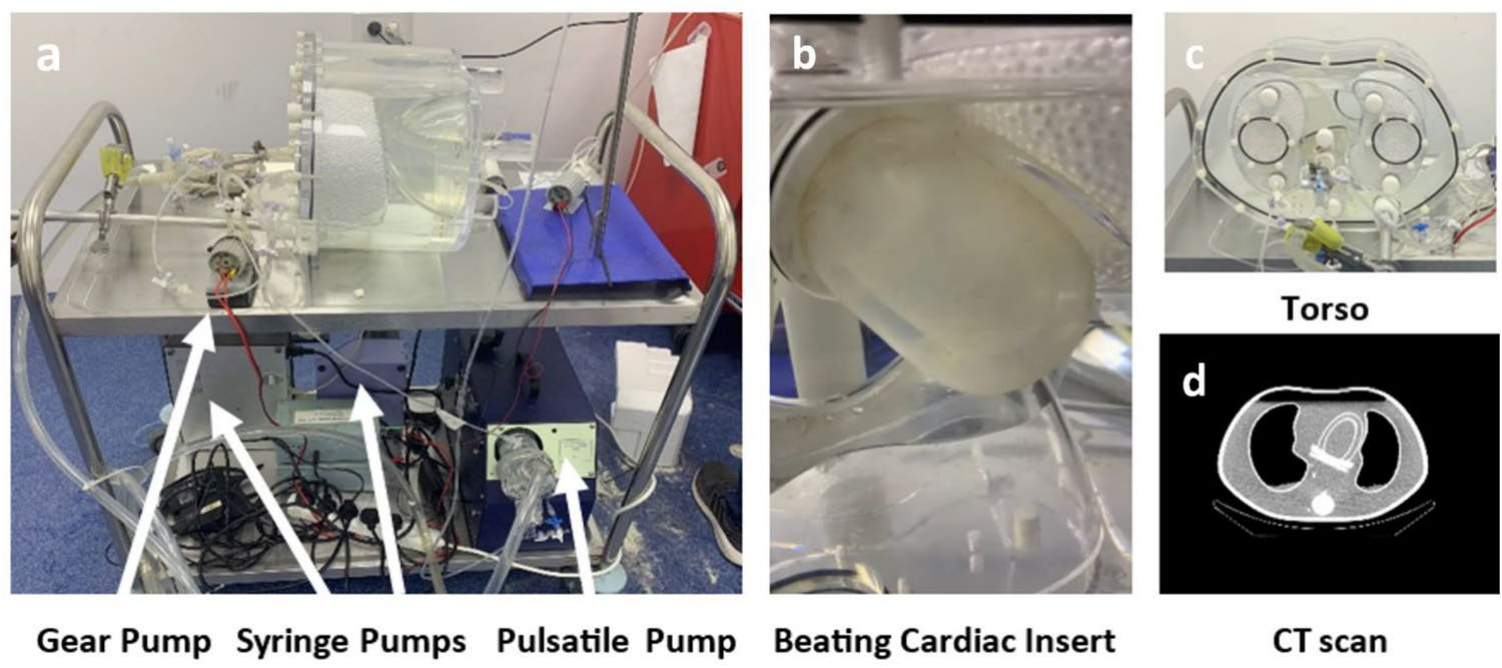
Anthropomorphic Beating Phantom. **a** Phantom and supportive pumps (pulsatile, syringe and gear), **b** Beating cardiac insert, **c** Phantom torso, **d** Phantom torso CT scan

The anthropomorphic phantom (Fig. 1) was described in detail previously [19, 20]. It consists of a beating cardiac insert placed in a commercial torso (Data Spectrum, Durham, NC, USA). The shape and dimensions of the insert are based on a commercial static cardiac insert (Cardiac Insert™, ECT/CAR/I, Data Spectrum) used for SPECT validation [26]. The beating feature is used during the entire acquisition and is allowed by flexible silicone membranes and a pulsatile pump (Pulsatile Blood Pump 55–3305, Harvard Apparatus, MA, USA) which can simulate heart rate and stroke volume by pumping a predefined volume of water in and out the LV compartment. The cardiac insert allows injection of a radiotracer like Tc-99m to its two compartments (LV and myocardium). In the LV compartment, it also allows washing out the radiotracer as occurs physiologically. In the myocardium compartment, after direct injection of the radiotracer in the initial wash in phase, the radiotracer stays in and circulates in a closed loop by a gear pump to ensure homogeneous distribution of the radiotracer in the myocardium region. The injection of the radiotracer into the two compartments is done by syringe pumps (Elite 11 Pump and PHD Ultra Pump, Harvard Apparatus, MA, USA). The concentration of the radiotracer for each compartment can be varied and adjusted per the MBF value to be tested.

The radiotracer concentration in each compartment, the parameters of the injection and washout protocols, and the actual concentrations in each compartment at the end of the experiment were used to create the LV and myocardium time activity curves using a previously published mathematical model [19].

The K1 was calculated from the TACs using the net retention model equation: ${K}_{1}=\frac{\frac{1}{{t}_{3}-{t}_{2}}{\int }_{{t}_{2}}^{{t}_{3}}{C}_{m}\left(t\right)-{S}_{m}{C}_{LV}\left(t\right)dt }{PV\times CF{\int }_{0}^{{t}_{1}}{C}_{LV}\left(t\right)dt}$ (1) where ${C}_{m}\left(t\right)$ and ${C}_{LV}\left(t\right)$ are the myocardial and LV radiotracer concentration activities. PV, ${S}_{m}$ and CF are the partial volume, spillover from LV to myocardium region and the myocardial density in g/ml. Integration limit ${t}_{1}$ denotes the end of the blood pool phase and ${t}_{2}$ and ${t}_{3}$ denote the integration limits of the average tissue activity. Default integration limits were used: ${t}_{1}={t}_{2}=1$ minute and ${t}_{3}= {t}_{2}+1$ minute. The MBF value was calculated using Renkin-Crone equation: ${K}_{1}=MBF\left(1-A{e}^{-\frac{B}{MBF}}\right)$ (2) where A = 0.874 and B = 0.443.

The injection and washout protocols were designed to generate TACs that resemble the TACs observed in human patients. The injection profile for the LV compartment included a short bolus (18 s) followed by a long washout (3 min) to flush the radiotracer. For the myocardium compartment, the radiotracer injection profile lasted 30 s initiated after the end of the LV injection. Both syringe pumps were programmed and synchronized using a computer with Harvard Apparatus’ dedicated software—FlowControl 1.0.5.

The radiotracer was injected into the LV and the myocardium compartment using 10 ml and 3 ml syringes correspondingly. The doses ranged between 3.2 to 6.4 mCi for the LV compartment and 1.5 to 6.4 mCi for the myocardium when most combinations were repeated 2 to 3 times. The administrated doses were chosen to reflect the number of counts in clinical setting [20]. The varied doses simulated a wide range of the MBF values (0.5–2 ml/min/g). This range covers entirely the normal rest values as well as the low part of the stress values. The pulsatile pump parameters (heart rate, stroke volume) were previously defined and described in detail [19, 20]. At the end of each experiment the phantom was stored for at least two days to avoid residual activity in the next experiment.

### StarGuide

**Fig. 2:**
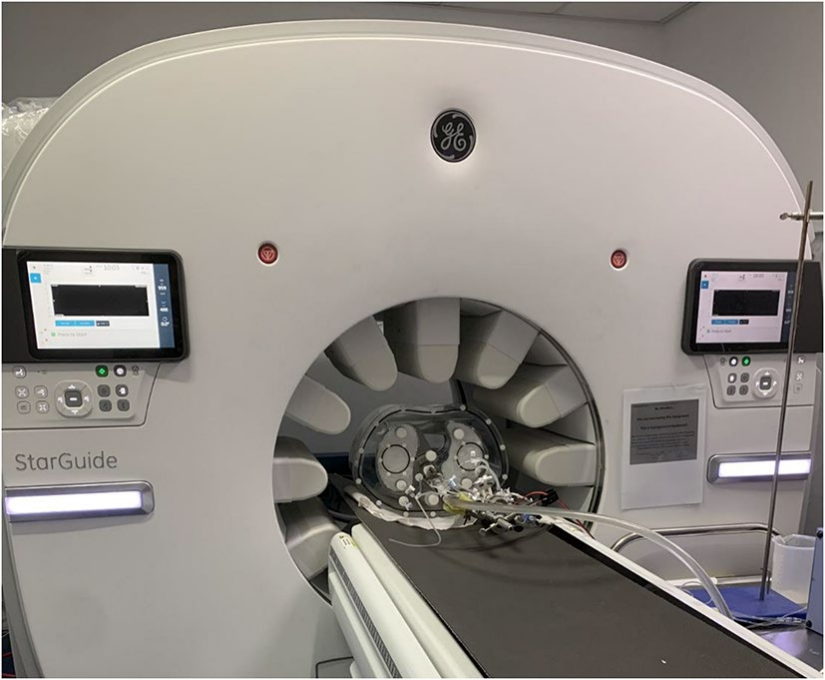
StarGuide system during phantom scan

The phantom was placed in the StarGuide system in supine, feet first position (Fig. 2). An automatic optical identification of the body contour, without the need for position dose, was performed by the system to plan the detectors’ motion and position during the scan. The acquisition was done using uniform mode. The scanning protocol length was 12 min divided into 48 consecutive, non-overlapping frames and 2 phases: 36 frames of 10 s each to capture the rapid changes following injection, followed by 12 frames of 30 s each for the steady state. Similar protocol with combinations of 10-s and 30-s frames was previously used [19, 20]. The system time resolution is 1 s so shorter frames could be defined and used. The sweep duration was set to 1 s. A short static scan of 6 min was followed as it was a required input for the 4DM software (for CFR calculation) but was not used in the analysis. The acquired data were processed, saved, and sent to the 4DM software (v2017, INVIA Medical Imaging Solutions, Ann Arbor, MI, USA) for TAC and MBF calculation. Attenuation correction was not applied. For tomographic reconstruction, only recorded events with energy of 140 keV ± 10% were considered. The reconstruction type was block sequential regularized expectation maximization (BSREM) with 10 iterations and 10 subsets. The penalty regularization function type was relative difference penalty (RDP) with penalty weight beta of 0.05. No post-reconstruction filtering was used. The same default parameters are used in clinical setting.

### 4DM software

**Fig. 3:**
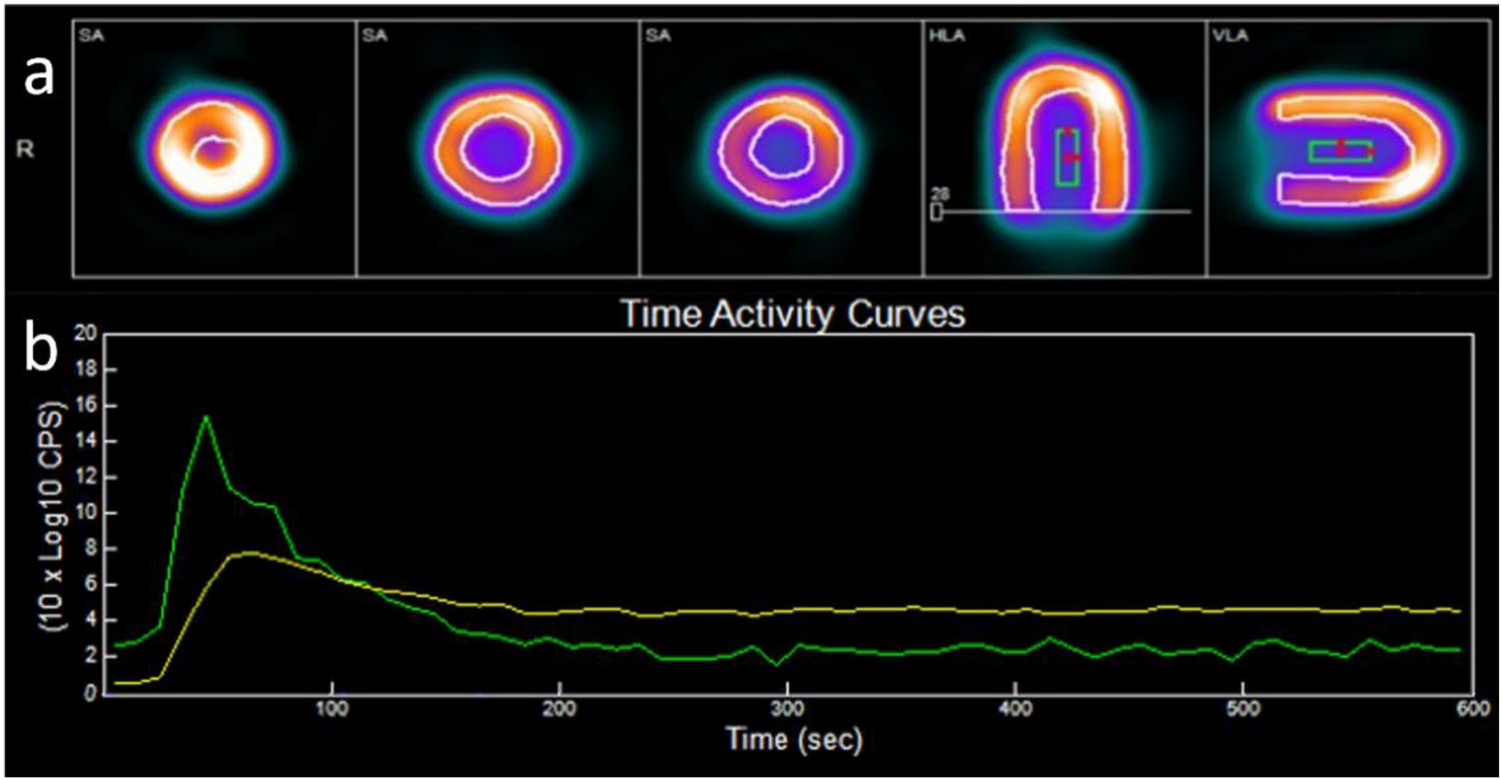
**a** 4DM segmentation and ROI location. **b** StarGuide TACs (green – LV TAC, yellow – Myocardium TAC)

The software analysis included review of the data and positioning of the region of interest (ROI) in the center of the LV compartment. An example of the software segmentation, ROI location and TACs provided in Fig. 3. The spillover from the LV to the myocardium ($${S}_{m})$$ was set to 0.25. It was calculated empirically by measuring the concentration level in the myocardium ($${C}_{m})$$ prior to the radiotracer injection to this compartment. At this early time, $${C}_{m}$$ is solely the result of the spillover from the LV compartment ($${C}_{LV})$$ and can be calculated using the equation: $${C}_{m}= {S}_{m}\bullet {C}_{LV}$$ (3). The PV value was 0.84 (default). The TACs were exported from the software and the MBF values were recorded for the statistical analysis. K1 was calculated from the MBF using Renkin-Crone Eq. (2).

### Statistical analysis

Pearson correlation was used to compare the phantom and StarGuide time activity curves (both LV and myocardium TACs). This was done for each experiment and the values were averaged. K1 and MBF derived from the TACs of the phantom and the StarGuide in each experiment were compared and averaged too. The difference, absolute difference and relative absolute difference were calculated. Mean and standard deviation were provided for each parameter. P-value and 95% confidence interval were calculated and P-value below 0.05 was considered significant.

## Results

**Fig. 4:**
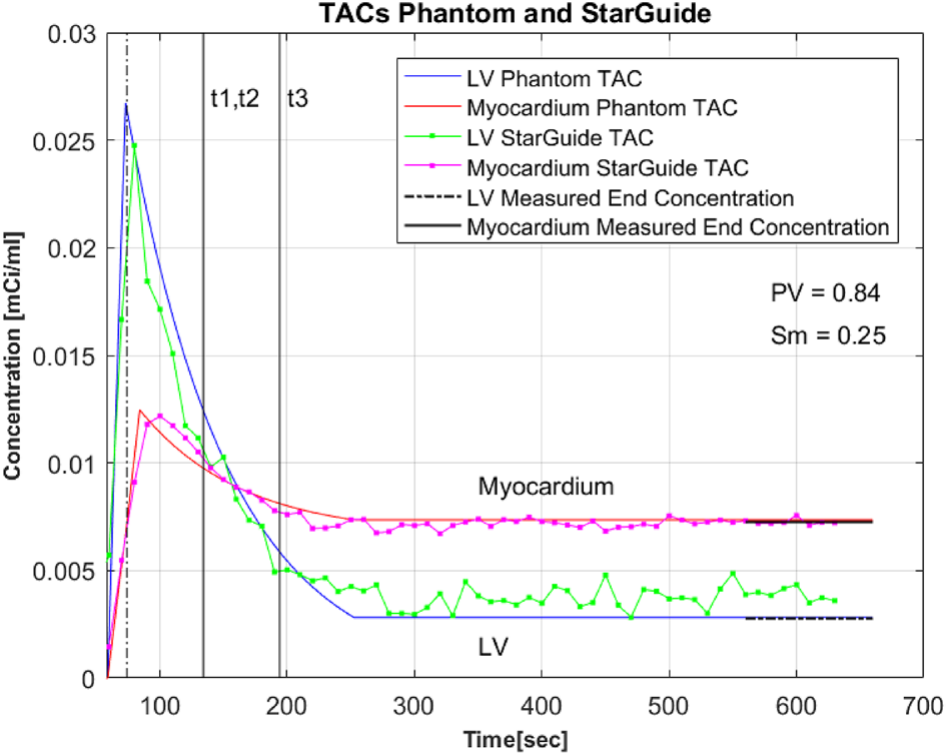
Example of TACs produced by the Phantom and StarGuide system TAC – time activity curve. LV – left ventricular. PV – partial volume. Sm – Spillover from LV volume to myocardium volume. t1 = t2 = 1 min after injection peak, t3 = t2 + 1 min (Eq. 1)

Fifteen experiments with various radiotracer concentrations representing different MBF values were conducted. The number of counts was similar to the one observed in a clinical setting [25]. The MBF values range was 0.47–1.83 ml/min/g. One experiment, due to injection of extremely high Tc-99m activity concentration, yielded a non-physiological MBF value of 6.97 ml/min/g. It was considered an outlier and excluded from the final analysis. An example of TACs calculated using the theoretical model of the phantom and measured by the StarGuide system is provided in Fig. 4.

The correlation between the TACs of the phantom and StarGuide was 0.94 ± 0.04, *p* < 0.001 for the LV and 0.89 ± 0.06, *p* < 0.001 for the myocardium. The K1 correlation between the phantom and the StarGuide was 0.984 [0.942–0.995], *p* < 0.0001 and the R^2^ was 0.969. The difference (${K1}_{Phantom}-{K1}_{StarGuide}$) was 0.02 ± 0.03. The relative difference $\frac{{K1}_{Phantom}-{K1}_{StarGuide}}{{K1}_{Phantom}}$ and the relative absolute difference $\frac{\left|{K1}_{Phantom}-{K1}_{StarGuide}\right|}{{K1}_{Phantom}}$ were 3.9 ± 6.3% and 6.2 ± 3.9% correspondingly.

**Fig. 5:**
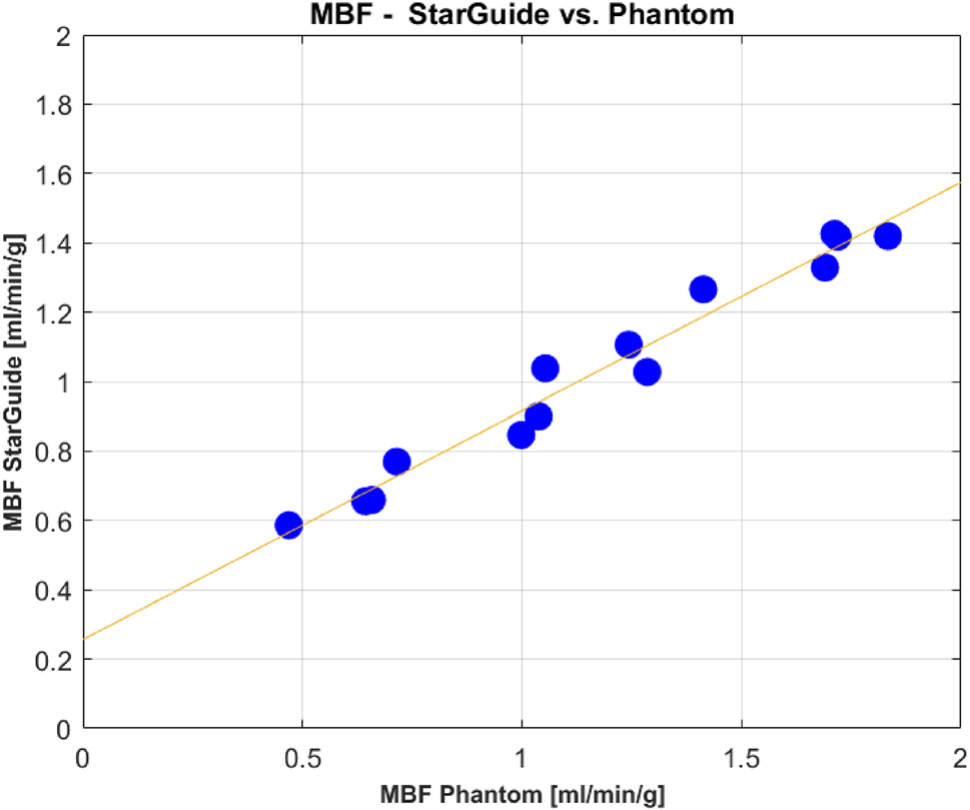
MBF values comparison – StarGuide system vs. phantom

**Fig. 6:**
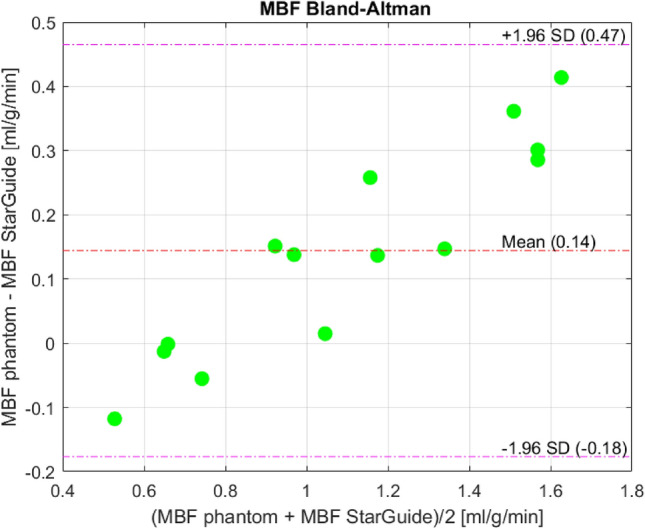
Bland–Altman plot of MBF values. SD – Standard Deviation

For the MBF, the correlation between the phantom and the StarGuide was 0.985 [0.951–0.995], *p* < 0.0001. The MBF difference was 0.14 ± 0.16 ml/min/g. The relative difference and the relative absolute difference were 8.2 ± 13.5% and 13.2 ± 8.1%. All the dynamic parameters appear in Table 1. The linear relationship between the phantom and the StarGuide MBF values is presented in Fig. 5. The difference between the MBF values was small for low values (near zero for MBF of 0.65) and it increased as the MBF value increased to 0.4 for MBF of 1.8. The Bland–Altman plot (Fig. 6) shows the distribution of the differences, which had a small mean (0.14 ml/min/g) and no outliers. A positive trend in the difference was observed. To further explore the error dispersion over the range, the MBF values were divided into 4 subranges (0.4–0.8, 0.81–1.2, 1.21–1.6, 1.61–2 ml/min/g) and the coefficient of variance (CV) was calculated. For the first three subranges, the CV was the same (11%) and for the last one it was lower (3%). The homogeneity of the regional uptake rate values was also tested by comparing the 3 main myocardium territories: left anterior descending (LAD), left circumflex artery (LCX) and right coronary artery (RCA) to the global uptake rate. The maximum difference was 5%.

**Table 1:**
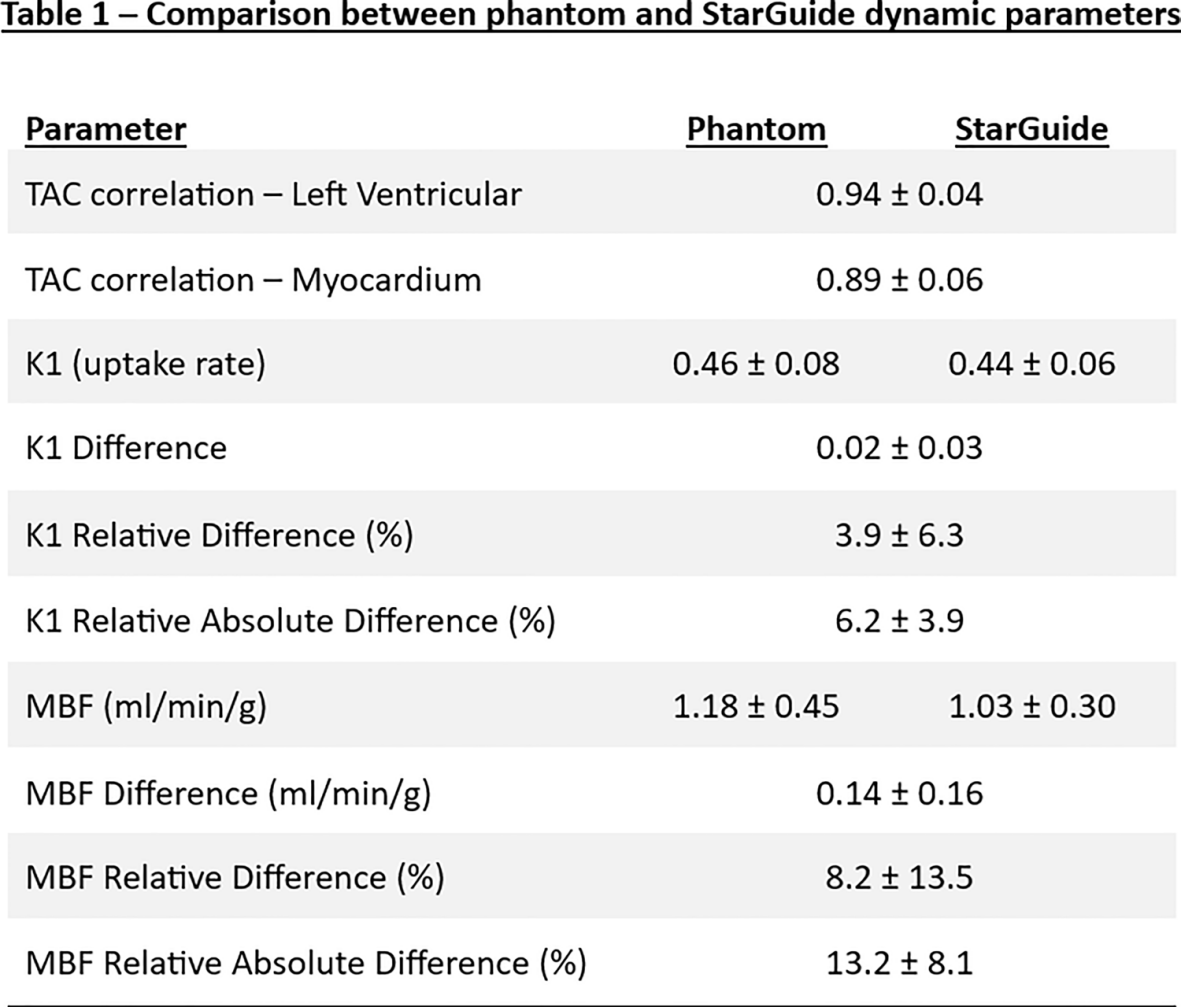
Comparison between phantom and StarGuide dynamic parameters

## Discussion

Our recently developed anthropomorphic beating phantom was used to validate the dynamic capabilities of the advanced StarGuide hybrid SPECT/CT system. For the validation, time activity curves were produced by the phantom which includes a novel two-compartment cardiac insert placed in a torso. A set of pumps allows the injection and washout of the Tc-99m radiotracer in a physiological manner while enabling a beating function. The StarGuide system acquired the emitted gamma ray photons using its CZT detectors and produced TACs using 4DM software which are expected to be similar to the phantom TACs. Fifteen experiments with various Tc-99m doses were conducted. The correlations between the phantom and the StarGuide were highly significant; 0.94 for the LV TAC and 0.89 for the myocardium TAC, *p* < 0.001, showing the high sensitivity of the StarGuide to track the physiological changes in the radiotracer concentration over time. Although a high correlation between the TACs was demonstrated, an overestimation during the steady state phase in the LV TAC of the StarGuide was observed. Exploring its source requires further investigation, however, in this study, it had no effect on the comparison of K1 and MBF between the phantom and the StarGuide, since the steady state phase is not included in the net retention model (see end of integration time – t3, in Fig. 4).

Using the net retention model, the uptake rate (K1) was derived from the TACs of each experiment. The correlation of the uptake rate between the phantom and the StarGuide was excellent (0.98, *p* < 0.0001). The difference in K1 value was small, 0.02 ± 0.03 and resulted in relative absolute error of 6.2 ± 3.9%. This mean relative error is very low considering experimental and technical uncertainties such as the Tc-99m activity concentration which was measured by a dose calibrator with accuracy of ± 2%. The uptake rates were converted to MBF values using the non-linear empirical Renkin-Crone Eq. (2). The conversion led to MBF difference of 0.14 ± 0.16 ml/min/g. The corresponding relative absolute error was 13.2 ± 8.1%, showing the high accuracy of the StarGuide system in assessing the MBF values.

As can been seen in the Bland–Altman plot, the difference between phantom MBF and StarGuide MBF values was small for lower values (around zero for MBF value of 0.65) and it increased on average when the MBF value was higher. In these high MBFs values, the StarGuide measurements overestimated the radiotracer concentration over time (the time activity curves) which led to an underestimation of the MBF value per Eq. 1. This might be related to the small difference between the radiotracer doses in the two compartments, LV and myocardium, in those experiments. Still, on average the MBF error was small (0.14 ml/min/g) showing the high accuracy of the StarGuide system.

Additional analysis of the error, in which the MBF values range was divided into 4 subranges (0.4–0.8, 0.81–1.2, 1.21–1.6, 1.61–2 ml/min/g) showed that although the error increases with MBF value, the dispersion in each subrange was good with a coefficient of variance of 11% or lower, demonstrating that StarGuide system provided precise MBF results. The regional uptake rate values of the 3 vessel territories were compared to the global value. As the radiotracer in the myocardium was well-distributed, a small difference was expected between the regions and indeed, the maximum difference was 5%, supporting the high precision of the system.

We previously used the phantom to validate the GE Discovery NM 530c camera [20]. When comparing our findings, we found similar results; low mean relative error (< 0.1 ml/min/g) and standard deviation (< 0.15 ml/min/g). This supports the accuracy of the new StarGuide system to assess MBF values. In addition, the regional homogeneity of the StarGuide system was better than the one observed for the Discovery NM 530c as the maximum uptake rate difference between the regional values and the global value was 5%, lower than the 13% previously reported for the Discovery NM 530c [20]. This improved homogeneity is attributed to the higher sensitivity of StarGuide system provided by its symmetrical ring of shape-adaptive detectors.

There is a wide range of clinical scenarios which can be tested using the novel phantom. This study tested a wide range of MBF values, covering both low and high values, but very high values reflecting stress scenario were not tested. The phantom can simulate higher MBF values (one MBF datapoint of 6.97 ml/min/g was an outlier and excluded from the analysis) and assessing those values is a subject of a future work. In addition, in this initial validation, the heart rate, the stroke volume, the ejection fraction and the injection rate were kept constant during the experiments and no defects were used to simulate ischemic areas. Therefore, the system was not tested in pathological scenarios such as low cardiac output with fluctuations. The novel phantom could also be used for validation of other systems and softwares. 4DM software was used in this study, but other software packages are available and could be tested and yield different findings. In addition, attenuation correction was not utilized in this study although the StarGuide system is a hybrid one and includes a CT scanner. This correction might improve the MBF results and could be examined using the phantom. An improved phantom design with longer lines and cables is needed to allow the acquisition of the CT scan and the SPECT scan during the same procedure. Other types of correction like scatter were not applied and therefore the focus of the analysis was global MBF values and not regional MBF which might require correction [20]. Respiratory correction was not needed as the current lungs are fixed. Attenuation and scatter corrections and the above-mentioned clinical and pathological scenarios are subjects for future research. Injecting positioning dose was not required when performing the experiments, as the StarGuide uses optical identification of the body contour. Without the positioning dose, the dynamic measurement is simpler and there is no need to subtract the background activity which could add to the inaccuracy of the measurement.

Accurate MBF assessment is highly valuable for diagnosis and prognosis and may result in better quality of care and improved clinical outcomes in patients with coronary artery disease. In this study, the advanced StarGuide hybrid SPECT/CT system with its ring array detector design and high temporal resolution, showed high accuracy and precision, comparable to the Discovery NM 530c, in assessing MBF values in comparison to an anthropomorphic beating phantom reference. The StarGuide system can reliably perform dynamic SPECT measurement over a wide range of myocardial perfusion flow rates.

## References

[1] Murthy VL, Bateman TM, Beanlands RS, Berman DS, Borges-Neto S, Chareonthaitawee P, et al. SNMMI cardiovascular council board of directors ASNC board of directors clinical quantification of myocardial blood flow using PET: joint position paper of the SNMMI cardiovascular council and the ASNC. J Nucl Med. 2018;59(2):273–93. 10.2967/jnumed.117.201368. 29242396 10.2967/jnumed.117.201368

[2] Camici PG, Rimoldi OE. The clinical value of myocardial blood flow measurement. J Nucl Med. 2009;50(7):1076–87. 10.2967/jnumed.108.054478. (**Epub 2009 Jun 12 PMID: 19525470**). 19525470 10.2967/jnumed.108.054478

[3] Valenta I, Dilsizian V, Quercioli A, et al. Quantitative PET/CT measures of myocardial flow reserve and atherosclerosis for cardiac risk assessment and predicting adverse patient outcomes. Curr Cardiol Rep. 2013;15:344. 10.1007/s11886-012-0344-0. 23397541 10.1007/s11886-012-0344-0

[4] Herzog BA, Husmann L, Valenta I, Gaemperli O, Siegrist PT, Tay FM, et al. Long-term prognostic value of 13N-ammonia myocardial perfusion positron emission tomography added value of coronary flow reserve. J Am Coll Cardiol. 2009;54(2):150–6. 10.1016/j.jacc.2009.02.069. (**PMID: 19573732**). 19573732 10.1016/j.jacc.2009.02.069

[5] Poitrasson-Rivière A, Moody JB, Renaud JM, Hagio T, Arida-Moody L, Buckley CJ, et al. Integrated myocardial flow reserve (iMFR) assessment: optimized PET blood flow quantification for diagnosis of coronary artery disease. Eur J Nucl Med Mol Imaging. 2023. 10.1007/s00259-023-06455-2. (**Epub ahead of print. PMID: 37807004**). 37807004 10.1007/s00259-023-06455-2 PMC11894525

[6] Patel KK, Spertus JA, Chan PS, Sperry BW, Al Badarin F, Kennedy KF, et al. Myocardial blood flow reserve assessed by positron emission tomography myocardial perfusion imaging identifies patients with a survival benefit from early revascularization. Eur Heart J. 2020;41(6):759–68. 10.1093/eurheartj/ehz389. (**PMID:31228200;PMCID:PMC7828468**). 31228200 10.1093/eurheartj/ehz389 PMC7828468

[7] Otaki Y, Manabe O, Miller RJH, Manrique A, Nganoa C, Roth N, et al. Quantification of myocardial blood flow by CZT-SPECT with motion correction and comparison with ^15^O-water PET. J Nucl Cardiol. 2021;28(4):1477–86. 10.1007/s12350-019-01854-1. (**Epub 2019 Aug 26. PMID: 31452085; PMCID: PMC7042031**). 31452085 10.1007/s12350-019-01854-1 PMC7042031

[8] Acampa W, Zampella E, Assante R, Genova A, De Simini G, Mannarino T, et al. Quantification of myocardial perfusion reserve by CZT-SPECT: a head to head comparison with ^82^Rubidium PET imaging. J Nucl Cardiol. 2021;28(6):2827–39. 10.1007/s12350-020-02129-w. (**Epub 2020 May 7 PMID: 32383083**). 32383083 10.1007/s12350-020-02129-w

[9] Cuddy-Walsh SG, deKemp RA, Ruddy TD, Wells RG. Improved precision of SPECT myocardial blood flow using a net tracer retention model. Med Phys. 2023;50(4):2009–21. 10.1002/mp.16186. (**Epub 2023 Jan 14 PMID: 36565461**). 36565461 10.1002/mp.16186

[10] Discovery NM530c and NM570c white paper. www.gehealthcare.co.uk

[11] Lee B, Ficaro E. Methods for the estimation of myocardial blood flow and coronary flow reserve with 99mTc SPECT in Corridor4DM. Ann Arbor: INVIA Medical Imaging Solutions; 2016.

[12] Gabrani-Juma H, Clarkin OJ, Pourmoghaddas A, Driscoll B, Wells RG, deKemp RA, et al. Validation of a multimodality flow phantom and its application for assessment of dynamic SPECT and PET technologies. IEEE Trans Med Imaging. 2017;36(1):132–41. 10.1109/TMI.2016.2599779. (**PMID: 28055829**). 28055829 10.1109/TMI.2016.2599779

[13] Bailliez A, Lairez O, Merlin C, Piriou N, Legallois D, Blaire T, et al. Left ventricular function assessment using 2 different cadmium-zinc-telluride cameras compared with a γ-Camera with cardiofocal collimators: dynamic cardiac phantom study and clinical validation. J Nucl Med. 2016;57(9):1370–5. 10.2967/jnumed.115.168575. (**Epub 2016 Apr 28 PMID: 27127220**). 27127220 10.2967/jnumed.115.168575

[14] Chrysanthou-Baustert I, Polycarpou I, Demetriadou O, Livieratos L, Lontos A, Antoniou A, et al. Characterization of attenuation and respiratory motion artifacts and their influence on SPECT MP image evaluation using a dynamic phantom assembly with variable cardiac defects. J Nucl Cardiol. 2017;24(2):698–707. 10.1007/s12350-015-0378-y. (**Epub 2016 Feb 4 PMID: 26846369**). 26846369 10.1007/s12350-015-0378-y

[15] Narihiro H, Masahisa O, Osamu H, Hiroyuki K, Masakazu M, Noriko M. Development of a 2-Layer double-pump dynamic cardiac phantom. J Nucl Med Technol. 2016;44(1):31–5. 10.2967/jnmt.115.168252. (**Epub 2016 Jan 14 PMID: 26769601**). 26769601 10.2967/jnmt.115.168252

[16] De Bondt P, Claessens T, Rys B, De Winter O, Vandenberghe S, Segers P, et al. Accuracy of 4 different algorithms for the analysis of tomographic radionuclide ventriculography using a physical, dynamic 4-chamber cardiac phantom. J Nucl Med. 2005;46(1):165–71 (**PMID: 15632048**). 15632048

[17] Debrun D, Thérain F, Nguyen LD, Léger CP, Visser JJ, Busemann-Sokole E. Volume measurements in nuclear medicine gated SPECT and 4D echocardiography: validation using a dynamic cardiac phantom. Int J Cardiovasc Imag. 2005;21(3):239–47. 10.1007/s10554-004-4014-1. (**PMID: 16015435**). 10.1007/s10554-004-4014-1 16015435

[18] Kamphuis ME, de Vries GJ, Kuipers H, Saaltink M, Verschoor J, Greuter MJW, et al. Development of a dedicated 3D printed myocardial perfusion phantom: proof-of-concept in dynamic SPECT. Med Biol Eng Comput. 2022;60(6):1541–50. 10.1007/s11517-021-02490-z. (**Epub 2022 Jan 19. Erratum in: Med Biol Eng Comput. 2022 Apr 8;: PMID: 35048275; PMCID: PMC9079041**). 35048275 10.1007/s11517-021-02490-z PMC9079041

[19] Krakovich A, Zaretsky U, Moalem I, Naimushin A, Rozen E, Scheinowitz M, et al. A new cardiac phantom for dynamic SPECT. J Nucl Cardiol. 2021;28(5):2299–309. 10.1007/s12350-020-02028-0. (**Epub 2020 Jan 29 PMID: 31997101**). 31997101 10.1007/s12350-020-02028-0

[20] Krakovich A, Zaretsky U, Gelbart E, Moalem I, Naimushin A, Rozen E, et al. Anthropomorphic cardiac phantom for dynamic SPECT. J Nucl Cardiol. 2023;30(2):516–27. 10.1007/s12350-022-03024-2. (**Epub 2022 Jun 27 PMID: 35760983**). 35760983 10.1007/s12350-022-03024-2

[21] Leppo JA, Meerdink DJ. Comparison of the myocardial uptake of a technetium-labeled isonitrile analogue and thallium. Circ Res. 1989;65(3):632–9. 10.1161/01.res.65.3.632. (**PMID: 2527638**). 2527638 10.1161/01.res.65.3.632

[22] Yoshida K, Mullani N, Gould KL. Coronary flow and flow reserve by PET simplified for clinical applications using rubidium-82 or nitrogen-13-ammonia. J Nucl Med. 1996;37(10):1701–12 (**PMID: 8862316**). 8862316

[23] Carsuzaa T, Thibault F, Bailly M. Gated tomographic radionuclide angiography using 3D-Ring CZT StarGuide SPECT/CT head-to-head comparison with a cardiac-dedicated CZT camera: first clinical use and validation. Clin Nucl Med. 2022;47(7):e515–7. 10.1097/RLU.0000000000004153. (**Epub 2022 Mar 31 PMID: 35353756**). 35353756 10.1097/RLU.0000000000004153

[24] Cerić Andelius I, Minarik D, Persson E, Mosén H, Valind K, Trägårdh E, et al. First clinical experience of a ring-configured cadmium zinc telluride camera: a comparative study versus conventional gamma camera systems. Clin Physiol Funct Imag. 2023. 10.1111/cpf.12853. (**Epub ahead of print. PMID: 37592454**). 10.1111/cpf.12853 37592454

[25] Bailly M, Callaud A, Metrard G. Dynamic cardiac SPECT with flow measurement using 3D-ring CZT: when SPECT is inspired by PET. Eur J Nucl Med Mol Imaging. 2023;50(6):1837–9. 10.1007/s00259-022-06106-y. (**Epub 2023 Jan 9 PMID: 36622405**). 36622405 10.1007/s00259-022-06106-y

[26] https://www.spect.com/pdf/cardiac-insert-with-fillable-solid-defect-sets.pdf

